# Brain Symmetry Index in Healthy and Stroke Patients for Assessment and Prognosis

**DOI:** 10.1155/2017/8276136

**Published:** 2017-01-30

**Authors:** Andrei Agius Anastasi, Owen Falzon, Kenneth Camilleri, Malcolm Vella, Richard Muscat

**Affiliations:** ^1^Department of Physiology & Biochemistry, Faculty of Medicine & Surgery, University of Malta, Msida, Malta; ^2^Centre for Biomedical Cybernetics, University of Malta, Msida, Malta; ^3^Neurology Department, Mater Dei Hospital, Msida, Malta

## Abstract

*Objective.* Quantitative neurophysiological signal parameters are of value in predicting motor recovery after stroke. The novel role of EEG-derived brain symmetry index for motor function prognostication in the subacute phase after stroke is explored.* Methods*. Ten male stroke patients and ten matched healthy controls were recruited. Motor function was first assessed clinically using the MRC score, its derivative Motricity Index, and the Fugl–Meyer assessment score. EEG was subsequently recorded first with subjects at rest and then during hand grasping motions, triggered by visual cues. Brain symmetry index (BSI) was used to identify the differences in EEG-quantified interhemispheric cortical power asymmetry observable in healthy versus cortical and subcortical stroke patients. Subsequently, any correlation between BSI and motor function was explored.* Results*. BSI was found to be significantly higher in stroke subjects compared to healthy controls (*p* = 0.023). The difference in BSI was more pronounced in the cortical stroke subgroup (*p* = 0.016). BSI showed only a mild general decrease on repeated monthly recording. Notably, a statistically significant correlation was observed between early BSI and Fugl–Meyer score later in recovery (*p* < 0.050).* Conclusions*. Brain symmetry index is increased in the subacute poststroke phase and correlates with motor function 1-2 months after stroke.

## 1. Introduction

Stroke is one of the leading causes of physical disability in adulthood, with more than a third of the 15 million yearly stroke sufferers being left with permanent disability [1]. Assessment of deficit and residual motor function in the clinical field is often restricted to bedside examination. The Medical Research Council (MRC) 0-to-5 scale muscle power assessment tool, originally designed for peripheral neuromuscular disorders, is one of the most ubiquitously used tools for motor power assessment in the clinical field [2]. More standardised semiquantitative scores, like the Motricity Index [3] and the Fugl–Meyer (FM) [4] scoring system, are limited to functional assessment in the research field. Prognostication of recovery is often based on the more simple bedside examination, along with the size of infarct on CT and MR imaging of the brain [5, 6]. Such benchmarks are often prone to interassessor variability and bias [2].

An increase in delta power (1–4 Hz) was one of the earlier features found to be associated with ischemic strokes [7]. This was later noted to decrease with improved recovery [8] and correlated with functional state and outcome [9]. The brain symmetry index (BSI) is one of the more popular EEG-derived parameters used in the research field for the purposes of stroke prognostication. It compares power spectra between the two cranial hemispheres and provides the magnitude of their asymmetry. First derived for detection of early brain ischemia during carotid surgery [10], its use has been extrapolated for the evaluation of ischemic changes after stroke [11–13].

The modified Rankin Scale (mRS), National Institutes of Health Stroke Scale (NIHSS), Glasgow coma scale (GCS), and acute physiology and chronic health evaluation II (APACHE II) are leading clinical tools which have been used as poststroke outcome measures and prognostic markers. Analyses of association between such clinical outcomes and EEG-derived parameters have shown significant correlation [14], suggesting promise for the role of such indices in the prognostication and management planning of patients after stroke.

This pilot study investigates the role of quantitative EEG parameters, particularly BSI, in the assessment and prognostication of motor function in the subacute phases following stroke. While several studies have used tools like the NIHSS as an outcome, few have focused more specifically on the motor sequalae following stroke. Rapid return of some motor function is often seen in the first days following stroke owing to resolution of temporary brain changes such as parenchymal oedema. This is, however, followed by a slower course of recovery along the following 6 months, attributed to neuroplasticity and reorganisation of brain networks. In light of this, repeated assessments were carried out along the first 4 months following stroke. In this pilot study, a longitudinal study of BSI and motor function along recovery could thus be carried out, having never been fully explored in literature so far.

## 2. Methods

### 2.1. Subject Recruitment

Twelve patients with first-ever stroke were initially recruited into the study. These had to be off antiepileptic medication, with no history of seizures, and fit enough to attend recording sessions outside of hospital. Recruitment was done in accordance with the Declaration of Helsinki and approved by the University of Malta Research and Ethics Committee (UREC). Patients had to be competent to understand and follow commands during sessions and also to sign an informed consent prior to recruitment.

Assessment sessions were carried out on a monthly basis at the University of Malta's Biomedical Engineering Laboratory. Data regarding patients' stroke imaging (CT or MR images) was obtained from Mater Dei Hospital after data protection approval. The only two female patients initially recruited for the study were later excluded from final analysis. The first female patient was excluded after the diagnosis of stroke was put into doubt in the context of normal imaging and noncharacteristic evolution of signs and symptoms, while the second female patient had to be excluded in view of poor cooperation to tasks during recording sessions. The demographic data and the status of the stroke lesions for the ten patients included in this study are shown in Table 1. Along with these ten male patients, ten healthy, age-matched male volunteers were recruited to act as controls and undergo the same assessment sessions. The average age of each group was 59.4 years and 60.3 years for stroke and healthy populations, respectively. Six of the ten male patients recruited agreed to be followed up with further neurophysiological and bedside motor function assessment. Out of the six patients followed up, three patients underwent a total of four assessment sessions (with the time from stroke reaching up to a maximum of 146 days) while the other three underwent a total of two sessions. The remaining four patients underwent one recording session before being lost to follow-up in view of comorbid conditions or other personal reasons. Four of the ten patients had cortical strokes while six were subcortical. Of the six patients who were followed up, half had cortical strokes while the other half had subcortical strokes.

### 2.2. Clinical Measures

A full neurological examination of the upper limb, with Medical Research Council (MRC) muscle power scoring, was conducted in the beginning of each session. The Motricity Index (MI) was then extrapolated by converting the MRC score to the equivalent MI subscores for (i) pincer grip, (ii) elbow flexion, and (iii) shoulder abduction sections, subsequently added together to give a single score for the upper limb, up to a maximum score of 100. The upper limb section of the Fugl–Meyer was thence obtained, giving a score up to a maximum of 66.

### 2.3. EEG Recording

After the clinical assessment, subjects were asked to sit in front of a laptop monitor and were fitted with a g.tec EEG cap with 32 Ag/AgCl electrodes referenced to one earlobe. The international 10–20 electrode placement standard, with higher concentration of electrodes in the central region over the motor cortex, was used. The EEG data was bandpass filtered using a Butterworth filter from 1 to 100 Hz and a notch filter was set at 50 Hz. EEG was recorded both at rest and during hand grasping motion. For the rest task (Task 1) subjects were asked to stay with their eyes open, but motionless, for 3 minutes, followed by a 3-minute period with eyes closed. For the hand grasping task (Task 2), subjects were asked to perform a hand grasping motion using a hand dynamometer while an LED light was on for a 5 s interval. This was repeated for 15 times. EMG data, force measurements, and other movement related potentials were also recorded and will be utilised for future analysis.

### 2.4. Data Processing

The EEG data was first rereferenced to a common average reference and a 1–25 Hz bandpass FIR filter was applied using forward-reverse filtering. Subsequently, data was epoched into 5-second trials with 30 epochs being acquired both during eyes open and during eyes closed. All trials and channels were manually reviewed and trials or channels with data consistently falling outside the 2 standard deviation range were excluded.

#### 2.4.1. The Brain Symmetry Index (BSI)

The brain symmetry index (BSI) was computed on background EEG obtained during Task 1, separated into periods with eyes open (BSI_o_) and eyes closed (BSI_c_), as well as on EEG data obtained during fist movement in Task 2, once again separated into BSI on EEG recorded from −2 s to onset of trigger (BSI_pre_) and EEG recorded during the first 2 s following the trigger instructing motion (BSI_post_).

A revised version of the BSI, developed by van Putten [15], was implemented in this study. This choice was based on its better sensitivity at detecting interhemispheric asymmetry [15, 16]. This was calculated on 11 centrally located electrodes on each hemisphere using the following equation:

(1) $$\begin{array}{c} BSI\left(\;t\;\right)=\frac{1}{K}\overset{K}{\underset{n=1}{\sum }}\left|\;\frac{R_{n}\left(t\right)-L_{n}\left(t\right)}{R_{n}\left(t\right)+L_{n}\left(t\right)}\;\right|, \end{array}$$

where *R*_*n*_(*t*) represents the average Fourier coefficient of 11 channels on the right hemisphere (after common average referencing) as calculated by *R*_*n*_(*t*) = (1/*C*)∑_*c*=1_^*C*^*a*_*n*_^2^(*c*, *t*), where *C* represents the number of channels (*C* = 11) and *a* represents the Fourier coefficient at channel *c* and at time *t* with a duration of *T* such that the time segment analysed lies within the range [*t* − *T*, *t*] (*T* was 5 s for BSI_c_ and BSI_o_ and 2 s for BSI_pre_ and BSI_post_). *K* represents the number of Fourier coefficients considered for the particular frequency band analysed [*k*_1_, *k*_2_] [15].

### 2.5. Data Analysis and Statistical Tests

**Figure 1:**
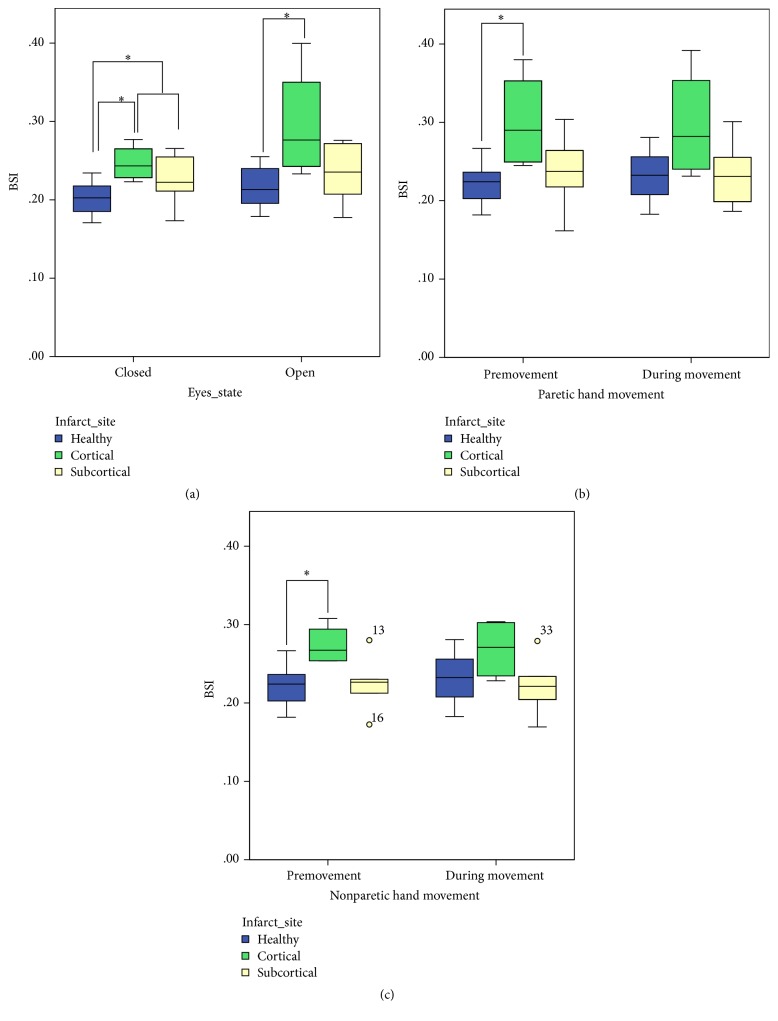
Brain symmetry index (BSI) scores obtained during (a) rest task (Task 1) with eyes either closed (BSI_c_) or open (BSI_o_) and during (b) paretic and (c) nonparetic hand grasping tasks (Task 2). Data is from session 1 of all 20 subjects, separated into healthy (blue), cortical (striped green), and subcortical (light yellow) stroke.

**Figure 2:**
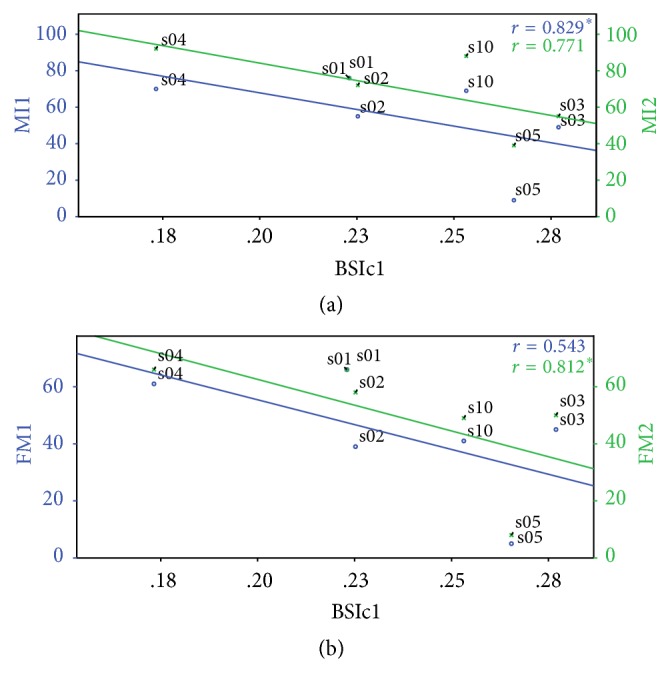
Correlation of brain symmetry index at rest recorded during the first session (BSI_c1_) with (a) Motricity Index in the first (MI1, blue) and second session (MI2, green) and with (b) Fugl–Meyer in the first (FM1, blue) and second session (FM2, green).

**Figure 3:**
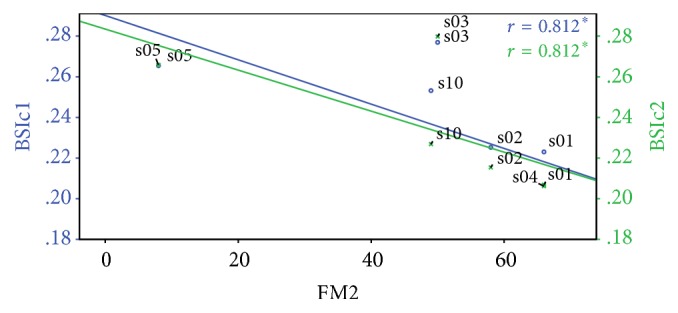
Correlation of Fugl–Meyer recorded in the second session (FM2) against original BSI in the first session (BSI_c1_, blue) and BSI recorded in the second session (BSIc2, green).

Data analysis was performed using the Statistical Product and Service Solution® (SPSS; v.20.0) software package for Windows®. Mann–Whitney *U* test was used to determine any statistically significant differences between population subgroup while Spearman's correlation was used to identify any significant correlation trends. The statistical significance level was set at *p* < 0.05. Asterisks in Figures 1–3 indicate which comparative analysis testing achieved this statistical significance level of *p* < 0.05.

BSI scores obtained in healthy subjects were compared with those of stroke subjects, the latter group being subdivided into cortical and subcortical strokes. This comparison was done using the Mann–Whitney *U* test. Subsequently, the BSI scores of stroke patients followed up with multiple sessions were correlated against time. Finally, the BSI scores obtained from stroke patients were correlated with their respective clinical motor scoring tools taken at various times during their recovery. This correlation was also carried out using the Spearmen correlation test.

## 3. Results 

### 3.1. Clinical Measurements for Motor Function Assessment

Information on session timing and corresponding functional status, based on Motricity Index (MI) [3] and Fugl–Meyer (FM) [4], is presented in Table 1. Upper limb function varied significantly in the patient group from almost complete resolution of symptoms in the first session (subject S09 with a maximum FM score of 66) to almost complete hemiplegia in the 4th session of testing (subject S05 with a FM score of 9 in his 4th session). Six stroke subjects were followed up with at least 2 sessions and all of these showed an overall improvement in upper limb function.

### 3.2. EEG Signal Data Analysis

#### 3.2.1. Brain Symmetry Index (BSI) in Stroke

Results of BSI obtained from EEG data recorded at rest are shown in Figure 1(a). A statistically significant difference was observed between the BSI obtained from healthy and stroke subjects (cortical and subcortical combined) with eyes closed (BSI_c_) (*p* = 0.023), where the BSI was noted to be higher in stroke subjects when compared to healthy controls indicating higher asymmetry in brain activity in the case of stroke patients. A similar difference was also noted in the BSI calculated with eyes open (BSI_o_) but not found to be statistically significant (*p* = 0.064). The BSI was also found to be even higher in cortical stroke, compared with subcortical stroke subjects. When healthy subjects were compared with cortical stroke patients, a statistically significant difference was also observed in both BSI_c_ and BSI_o_ (*p* = 0.016 and *p* = 0.028, resp.).

The BSI was also calculated from the EEG data obtained during Task 2, both during the actual hand grip motion (BSI_post_) and also just prior to each movement (BSI_pre_). Figures 1(b) and 1(c) show the median and quartile ranges of BSIs during different conditions related to Task 2, looking at EEG data within the 1–25 Hz. Healthy control subject H09 had to be excluded from the EEG analysis during movement trials in view of significant artefacts interfering with data.

Significant disparity between healthy and cortical stroke subjects was found when BSIs were calculated from EEG data just prior to paretic and nonparetic hand movement, respectively (BSI_pre_). This is consistent with the statistically significant differences previously obtained with eyes open and eyes closed EEG, showing higher BSI scores in stroke patients when compared to healthy controls (*p* = 0.031). The BSI calculated during actual movement (BSI_post_) showed no statistically significant differences between the healthy and any of the stroke subgroup.

#### 3.2.2. Change in BSI with Time

In the six stroke subjects who were followed up with at least two sessions, a general decrease in BSI was observed with time but this trend was not consistent in all subjects. No statistically significant correlation was found between BSI scores (BSI_o_ and BSI_c_) and session number (1 to 4) (*r* = −0.427, *p* = 0.077 and *r* = −0.320, *p* = 0.196, resp.).

#### 3.2.3. Correlation of BSI with Motor Function

The level of correlation between the brain symmetry index calculated from EEG data at rest and the motor function as assessed by clinical scoring tools (Motricity Index (MI) and Fugl–Meyer (FM)) performed in session 1 and session 2 for each patient followed up was subsequently computed as shown in Table 2. BSI_o_ showed no significant correlation with any functional scores and thus only results involving BSI_c_ will be presented below and in Table 2.

BSI_c_ scores obtained during the first session (BSI_c1_) were compared to the clinical scores taken concurrently in the same session for all ten stroke patients. Neither the Fugl–Meyer score in the first session (FM_1_) nor the Motricity Index in the first session (MI_1_) achieved statistically significant correlation levels when compared to BSI_c1_ (when all ten patients where considered). A statistically significant negative correlation was observed between the Motricity Index and BSI_c1_ session from the six patients who were followed up (*r* = −0.829, *p* = 0.042) as seen in Figure 2(a). The correlation between Fugl–Meyer and BSI_c1_ in this subgroup did not reach statistical significance (*r* = −0.543, *p* = 0.266) as shown in Figure 2(b).

Nevertheless, when Fugl–Meyer scores recorded in the second session (FM_2_) were compared with BSI_c1_ (i.e., acquired in the first session), a statistically significant negative correlation was notably observed (*r* = −0.812, *p* < 0.05) (Figures 2(b) and 3). This represents a possible prognostication value for the score in question: BSI_c_ calculated from an early EEG after stroke is here shown to be correlated with functional motor outcome about 2 months down the line, as quantified by Fugl–Meyer scoring. This significance is sustained when FM_2_ were compared with the BSI_c_ in the second session (BSI_c2_) (*r* = −0.812, *p* < 0.05) (Figure 3). However, no further correlations were found between BSI_c2_ and functional scores and so the correlation tests between BSI_c2_ and clinical measures are not included in Table 2.

**Figure 4:**
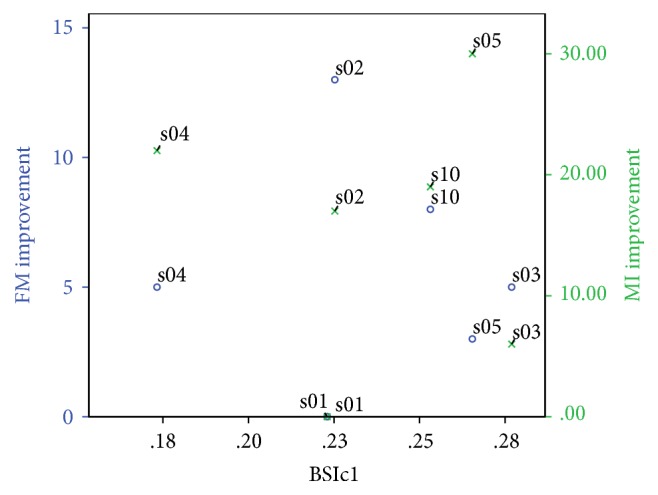
FM and MI improvement versus BSIc1 for patients who had at least 2 assessment sessions.

**Figure 5:**
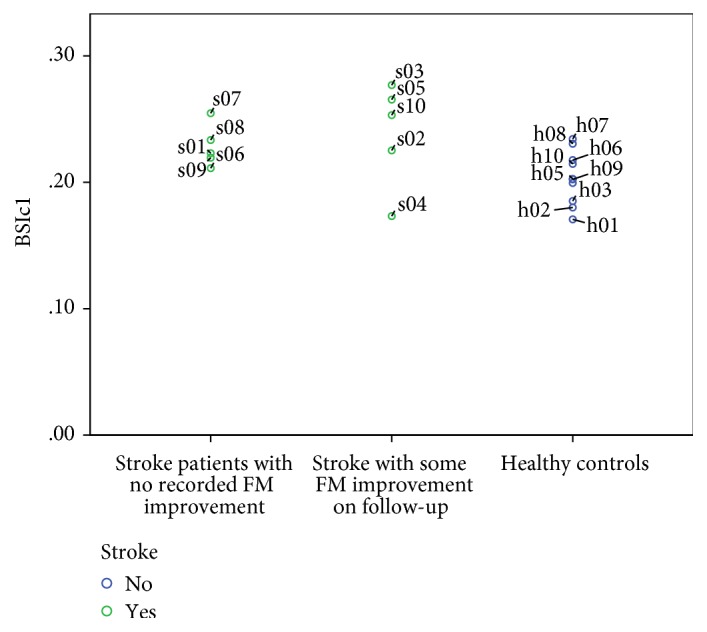
BSIc1 of all 20 subjects recruited. “Stroke patients with no recorded FM improvements” may be either due to lack of follow-up (s06–s09) or because FM1 was already 66 (s01).

**Figure 6:**
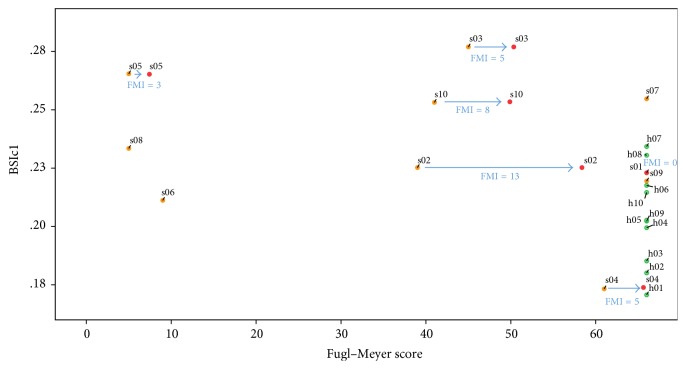
Brain symmetry index in session 1 (BSIc1) from all 20 subjects, against their Fugl–Meyer score both in session 1 (yellow bubble for stroke patients and green bubble for healthy controls) and in session 2 (red bubble) when this was present. The blue arrow and score represents the Fugl–Meyer improvement (FMI) for those who had a second (follow-up) session.

Fugl–Meyer and Motricity Index improvement from session 1 to session 2 for the six patients who managed to attend the follow-up sessions was also calculated and plotted with BSIc1 (Figure 4). However, in view of the fact that some patients started with a very high clinical score in session 1 (for example S01 with FM-66), this meant that the specific clinical score improvement was not representative of a good clinical outcome in these patients. Therefore, final Fugl–Meyer was taken as a measure of good positive outcome rather than improvement.  Figure 5 illustrates BSc1 of all 20 subjects against both FM1 and FM2 when this was available.  Figure 6 on the other hand illustrates BSc2 of all subjects divided into healthy, stroke with FM improvement, and stroke without FM improvement (either due to lack of follow-up or because FM1 was already maximum at initial visit).

## 4. Discussion

### 4.1. Clinical Measurements, Outcomes, and Patient Experience

Significant heterogeneity in stroke type and extent of disability following the insult were observed in our study population. All the six patients who were followed up improved to some extent in upper limb function. In the clinical setting, this is often defined using MRC scoring, while, in the research field, more comprehensive clinical scoring tools are used as standards, with the Fugl–Meyer being a leading one. This study successfully presents a very strong correlation between the Fugl–Meyer and the MRC score, along with its derivative, the Motricity Index. This supports the comparability and validity of using both assessment tools in motor function assessment at the bedside. Nevertheless, correlation between clinical scores and neurophysiological signal parameters was stronger with Fugl–Meyer than with MRC and Motricity Index scores, reflecting the more detailed and accurate nature of the Fugl–Meyer over the other two time-efficient bedside scores. The superiority of the Fugl–Meyer score in this context supports its use in studies on motor function and neurophysiological signal analysis [17, 18] as well as its use in the clinical field.

Over the four-month period of follow-up, no major health complications were seen in the study population, reflecting the recruitment bias inclined towards healthier patients fit enough to attend sessions outside hospital. All three subjects who were followed up with the maximum of 4 sessions claimed they felt a positive, beneficial effect following each of the sessions, in spite of the lack of intervention implemented. Such subjective positive effect may have been secondary to the focused movement tasks applied during the assessment, the online biofeedback reflecting the patients' force applied, and the presentation of objective evidence of improvement to each patient after each session.

### 4.2. EEG Signal Data Analysis

#### 4.2.1. Brain Symmetry Index (BSI) in Stroke

Early BSI measurement from continuous EEG recorded in the first few hours to days following stroke has been significantly explored in literature [11, 14]. This study, however, observes BSI measures in the subacute phase following stroke, starting on average at 26 days, and extended the longitudinal analysis over a longer period of time. Considering first the BSI calculated at rest with eyes closed, a significantly higher level of asymmetry was observed in stroke subjects (corresponding to a higher BSI, with a *p* value of 0.023), which is consistent with previous studies using this index [11]. This represents the focal disruption that arises in one hemisphere following stroke. A stronger statistical significance was observed when specifically cortical stroke subjects were compared with healthy controls. These exhibited even higher BSIs (*p* value of 0.016). This larger asymmetry from cortical stroke patients is expected considering that EEG recordings are more representative of superficial cortical electrical activity than deeper subcortical activity. For this reason BSI values acquired from patients with subcortical (or deep) infarcts are significantly closer to those of healthy patients. In spite of the less pronounced effect of subcortical infarcts on EEG, they are known to be particularly prone to lead to significant clinical signs and symptoms in view of the concentration of neural tissue that is found within the deeper structures of the brain.

Interestingly, differences in asymmetry between stroke and healthy subjects were less pronounced when subjects were asked to stay at rest with their eyes open, as opposed to their eyes being closed. Most studies in fact analyse EEG only during the eyes closed state [9, 19], one of the reasons being to reduce the amount of artefacts (including eye blink artefacts) that interfere with EEG [14]. Possible reasons for the discrepancy in statistical significance achieved between the two states in our results are various: the presence of a larger number of artefacts present while a subject has his eyes open may broaden the range of power distribution recorded across the different trials thence increasing the variance of results within each group and decreasing the power and significance of any comparative studies. Furthermore, upon eye closure, suppression of symmetrical cortical activity like that found in the occipital cortex may have an influence on the BSI, which does not have significant motor cortex selectivity.

BSI calculated during motor imagery has been shown to correlate with function and Fugl–Meyer improvement in Ang et al. [16]. In our study, BSI was calculated during actual movement yet still no statistically significant difference in BSI was observed between different subject groups. Only the BSI captured immediately prior to each movement (within the same movement trials of Task 2) exhibited a statistical significant difference between cortical stroke patients and healthy patients, which complements the higher BSI observed in cortical stroke patients in background EEG captured at rest. The difference in statistical significance obtained between the two studies during movement might stem from the more robust robotic exoskeleton-controlled setup employed in Ang et al. [16] study which eliminates several confounding movements.

#### 4.2.2. Change in BSI with Time

Although there was an overall decline in BSI scores from the first to the last session, no statistically significant trend was shown and no significant correlation between BSI scores and session number was found. This can be partly attributed to the limited amount of follow-up that was achieved in the stroke subgroup, limiting the power of such correlation analysis.

#### 4.2.3. Correlation of BSI with Motor Function

Correlation of BSI with functional status and eventual outcome have been the subject of great interest in research. Higher BSIs have consistently been associated with higher NIHSS scores on admission, higher disability in the following months, and lower survival rates [11–14]. This study focuses purely on the recovery of motor function as opposed to broader morbidity and mortality outcomes. For the purposes of quantifying motor function and outcome, the Medical Research Council (MRC) muscle power scale from which the Motricity Index (MI) was derived, along with the Fugl–Meyer (FM) assessment tool, was used.

The level of correlation between function (FM and MI) and BSI during an individual session was first determined for all ten subjects in the patient subgroup. To our knowledge, correlation of Fugl–Meyer with BSI has only been explored in literature as a tool for quantifying the benefits of a BCI robotics therapy in a placebo-controlled trial [16] (significant inverse correlation was reported between a BSI and Fugl–Meyer outcome after intervention in this context). In contrast, this study explores the relationship between Fugl–Meyer and BSI scores along the course of recovery in patients following conventional physiotherapy and occupational therapy-based rehabilitation programs, for the purposes of prognostication rather than assessment of benefit from any particular intervention.

From results presented earlier in this paper, the most notable finding was the statistically significant negative correlation observed between initial BSI score and functional motor outcome, as measured by Fugl–Meyer score during follow-up sessions in the study. In other words, higher BSI values in the first session were associated with lower final Fugl–Meyer scores recorded in the second session and hence a poorer functional outcome, with a correlation significance of just below *p* = 0.05. This has great implications with respect to the role of BSI in prognostication and prediction of motor function. Correlation at this level implies that quantitative EEG parameters measured early in stroke could possibly aid in prognostication of functional outcome months down the line. Statistical testing on data acquired during third and fourth follow-up sessions was not carried out due to the very limited amount of patients who were followed up for this length of time, making statistical analysis impractical.

Correlation analysis between BSI and clinical scoring tools in the first session did not give statistically significant results in this study. The reason for the lack of a correlation between FM_1_ and BSI_1_, in the presence of a significant correlation between FMI_2_ and BSI_c1_ and between FM_2_ and BSI_c2_, is worth considering. One possible reason for this discrepancy is the fact that motor function and deficit in the first 4 weeks after stroke (coinciding with the timing of the first session in our study) is multifactorial and can depend on temporary phenomena like intraparenchymal vascular oedema, reversible ischemia, and other fluctuating brain changes that do not necessarily improve in a predictable or linear fashion with time. Beyond this acute period, a more steady and gradual course of improvement in function is expected secondary to neuroplasticity, which is more closely linked to the true extent of the initial brain insult sustained. Neurophysiological indices acquired early following the insult, like the BSI_1_, could be selectively capturing areas of neurophysiological changes within the hemispheres that are more substantial and well established (over the more temporary factors mentioned above) and that thus correlate with stroke extent and eventual recovery once the acute period has subsided.

### 4.3. Limitations

The study design employed for this pilot study was that of a prospective case-control study based on identical assessment tools in each group and without any intervention introduced on either group of patients. A significant limitation to the power of the results lies in the small number of patients included in the study. Challenges to recruitment and inclusion of patients were based on small population and tight exclusion criteria, where patients had to be fit enough to be transported out of the medical facility to a dedicated research EEG lab and comply with the tests for 1 to 2 hours at a time. Similarly, the high rate of drop-outs limited the amount of statistical analysis possible with respect to longitudinal changes in EEG patterns and motor function with recovery. Nevertheless, with the limited amount of subjects recruited and the age and sex matched control population studied, significant results could still be achieved, showing promise for larger studies in the near future.

Technical limitations which interfered with robustness of EEG recording during movement is the lack of exoskeleton-based motor restraint which, as mentioned previously, would allow for a more selective analysis of specific muscle movements and decrease unwanted confounding movements.

### 4.4. Conclusions

Quantitative assessment and prognostication of motor function following stroke is still sparse in the clinical field and has become the subject of a growing research interest in neurorehabilitation. Several technology-based rehabilitation solutions are currently being investigated yet the heterogeneity of stroke pathology and recovery patterns makes standardisation of novel therapies problematic. The use of neurophysiological and imaging techniques for the purposes of motor recovery prediction has been suggested to be beneficial in prescribing patient-specific rehabilitation programs and goals [20].

EEG parameters like the brain symmetry index (BSI) have been used in research as a prognostic marker for general morbidity and mortality following stroke, yet the role of BSI in functional motor recovery has never been considerably explored before. In this work, a prospective case-control study involving ten patients suffering from stroke along with ten healthy matched controls has been undertaken and the outcomes obtained show that there exists a correlation between EEG parameters and patients' functional motor outcome during stroke recovery.

Results from this study support the utility of the BSI utility as a prognostic marker for motor recovery. Initial analysis comparing the BSI scores of healthy and stroke subjects confirmed a higher asymmetry following stroke, most identifiable from EEG data acquired at rest and just prior to movement. A longitudinal analysis of change in BSI across the first few months following stroke (a temporal analysis which has been scarcely conducted in the context of BSI in previous literature) did not produce statistically significant trends of change along recovery. Nevertheless, analysis of change in motor function in relation to the BSI produced an inverse correlation between follow-up Fugl–Meyer and initial BSI, making the role of BSI as a prognostic marker of motor function and outcome promising.

## Figures and Tables

**Table 1:** Demographic and clinical data of stroke patients included in study.^1^

Subject	Age	Infarct site	Session timing and motor function			
			Session 1	Session 2	Session 3	Session 4
S1	65	CS, left middle cerebral artery territory	Poststroke: 31 days **MI: 76** **FM: 66**	Poststroke: 60 days **MI: 76** **FM: 66**	Poststroke: 92 days **MI: 76** **FM: 66**	Poststroke: 119 days **MI:** **100** **FM: 66**
S2	67	SCS, right capsular	Poststroke: 5 days **MI: 55** **FM: 39**	Poststroke: 43 days **MI: 72** **FM: 58**	Poststroke: 63 days **MI: 72 FM: 61**	Poststroke: 92 days **MI: 76** **FM: 64**
S3	69	CS, left middle cerebral artery territory	Poststroke: 14 days **MI: 49** **FM: 45**	Poststroke: 31 days **MI: 55** **FM: 50**		
S4	58	SCS, right side of pons	Poststroke: 7 days **MI: 70** **FM: 61**	Poststroke: 58 days **MI: 92** **FM: 66**		
S5	70	SCS, left basal ganglia	Poststroke: 38 days **MI: 9** **FM: 5**	Poststroke: 87 days **MI: 39** **FM: 8**	Poststroke: 112 days **MI: 47** **FM: 10**	Poststroke: 146 days **MI: 39** **FM: 9**
S6	47	SCS, posterior limb of right internal capsule	Poststroke: 12 days **MI: 22** **FM: 9**			
S7	63	SCS, right basal ganglia	Poststroke: 60 days **MI: 100** **FM: 66**			
S8	39	CS, right middle cerebral artery	Poststroke: 33 days **MI: 23** **FM: 5**			
S9	52	SCS, left basal ganglia	Poststroke: 16 days **MI: 92** **FM: 66**			
S10	64	CS, right pre- and postcentral gyri	Poststroke: 41 days **MI: 69** **FM: 41**	Poststroke: 91 days **MI: 88** **FM: 49**		

^1^CS: cortical stroke; SCS: subcortical stroke; MI: Motricity Index (maximum score 100); FM: Fugl Meyer (maximum score 66).

**Table 2:** Correlation between BSI in the first session (BSI_c1_) and clinical measures FM and MI.

BSI	Clinical score	Spearman correlation
BSI_c1_	FM_1_	*r* = −0.543, *p* = 0.266
	FM_2_	*r* = −0.812, *p* < 0.050
	MI_1_	*r* = −0.829, *p* = 0.042
	MI_2_	*r* = −0.771, *p* = 0.072
